# Diet and Pancreatic Cancer Prevention

**DOI:** 10.3390/cancers7040892

**Published:** 2015-11-23

**Authors:** Ilaria Casari, Marco Falasca

**Affiliations:** Metabolic Signalling Group, School of Biomedical Sciences, CHIRI Biosciences, Curtin University, Perth 6102, Australia; ilaria.casari@curtin.edu.au

**Keywords:** pancreatic cancer, cancer prevention, diet, obesity

## Abstract

Pancreatic cancer is without any doubt the malignancy with the poorest prognosis and the lowest survival rate. This highly aggressive disease is rarely diagnosed at an early stage and difficult to treat due to its resistance to radiotherapy and chemotherapy. Therefore, there is an urgent need to clarify the causes responsible for pancreatic cancer and to identify preventive strategies to reduce its incidence in the population. Some circumstances, such as smoking habits, being overweight and diabetes, have been identified as potentially predisposing factors to pancreatic cancer, suggesting that diet might play a role. A diet low in fat and sugars, together with a healthy lifestyle, regular exercise, weight reduction and not smoking, may contribute to prevent pancreatic cancer and many other cancer types. In addition, increasing evidence suggests that some food may have chemo preventive properties. Indeed, a high dietary intake of fresh fruit and vegetables has been shown to reduce the risk of developing pancreatic cancer, and recent epidemiological studies have associated nut consumption with a protective effect against it. Therefore, diet could have an impact on the development of pancreatic cancer and further investigations are needed to assess the potential chemo preventive role of specific foods against this disease. This review summarizes the key evidence for the role of dietary habits and their effect on pancreatic cancer and focuses on possible mechanisms for the association between diet and risk of pancreatic cancer.

## 1. Introduction

Pancreatic cancer is an aggressive disease which holds the gloomy record of having become one of the most deadly malignancies in the USA, being the fourth leading cause of cancer-related death in the USA, despite holding the 10th place in the incident rate scale [1,2]. Due to the difficulty in obtaining an early diagnosis and to its resistance to treatment, pancreatic cancer has a very poor prognosis, with a five-year survival rate of 7%, according to the American Cancer Society. Although recent studies have pointed out the diversity and complexity of pancreatic cancer genetics, some predisposing factors have been identified for this disease. Genetic factors are the main overt factors responsible, followed by other DNA damage-inducing causes such as age, smoking (implicated in 20%–25% of cases), type 2 diabetes and chronic pancreatitis [3,4]. However, dietary components are also thought to play a part in the development of this disease, as obesity, and high consumption of red meat and fried foods are all risk factors. Conversely and according to some studies, a diet rich in vegetables, fresh fruit, nuts and whole grain is useful in the prevention of pancreatic cancer [1,5,6,7]. In this review, we will discuss the possible links between diet and pancreatic cancer, analysing the potential role of diet in promoting or preventing the onset of this disease.

## 2. Pancreatic Cancer Progression Model

The pancreas is an elongated organ situated behind the stomach that holds digestive and hormonal functions. Digestive enzymes are secreted by its exocrine gland, while the islets of Langerhans, which constitute the endocrine gland, are devolved to hormones secretion. The vast majority of tumours originate in the exocrine gland and, amongst them, 90% are pancreatic ductal adenocarcinoma (PDAC), which is also the most aggressive form of pancreatic cancer. Due to the fact that, especially in its early stages, pancreatic cancer can be asymptomatic or associated to non-specific symptoms (they may include nausea, indigestion, weight loss, back pain, abdominal pain, jaundice, steatorrhea and depression), it is rarely possible to obtain an early diagnosis and this therefore implies an even greater difficulty of treatment [8]. The development of PDAC is very complex and it is characterized by a sequence of precursor lesions, which occur at different stages of time and ultimately evolve into invasive cancer. These lesions are known as pancreatic intraepithelial neoplasia (PanIN), intraductal papillary mucinous neoplasms and mucinous cystic neoplasm. The majority of PDACs develop from PanINs, and have been classified according to their morphological, cytological, and genetic transformations, each of which leads to significant alterations of the signalling pathway. Three stages, characterized by increasingly atypical cells, have been identified and called PanIN1 (sub classified into PanIN1a and PanIN1B), PanIN2 and PanIN3 [9]. One of the early events in the progression of the disease is the occurrence of Kras gene mutations at codon 12 in the normal pancreatic epithelial cells, which are a characteristic of 90% of all PDAC. These mutations induce cellular proliferation, invasion and survival. In the intermediate stage of the disease, the inhibition of the p16 tumour suppressor gene occurs, promoting further cytological and architectural atypia in the duct cells. In a later stage, the inactivation of the p53 tumour suppressor gene (TP53), which is normally responsible for DNA repairing, cell division blocking and apoptosis activation, takes place in 70% of all PDAC. In the late stage of the disease, inactivation of the SMAD4 gene involved in the cell signalling pathway also occurs in 55% of all PDAC [10].

### PDAC Defining Features

PDAC defining features can be summarized in: altered metabolism, desmoplasia, and hypo vascularization. In order to survive and proliferate in the new micro-environment, pancreatic cancer cells are forced to be subjected to a metabolic reprogramming and they rely on anabolic reactions to synthetized *de novo* proteins, nucleic acids and lipids. Oncogenic Kras has been found to play a key part in PDAC metabolism rearrangement. One of the most important constituents of pancreatic cancer cells metabolism is autophagy. Autophagy is a very important catabolic process, regulated by several protein complexes, whose function is to recycle unneeded or damaged cellular particles, protein or molecular complexes, in order to maintain cells homeostasis. This function is also anti-tumorigenic as it contributes to control pro-tumorigenic elements such as tissue damage, oxidative stress and genomic imbalance. On the contrary, when increased above the baseline in established tumours, autophagy becomes a pro-tumorigenic factor by furnishing cancer cells with nutrients and energy [11]. Desmoplasia, which is another key feature of PDAC, is a complex reaction of pancreatic stroma that involves stroma components such as stellate cells, leukocytes, endothelial cells, fibroblasts, and extracellular matrix, as well as invading tumour cells and growth factors, and results in fibrotic tissue formation. The characteristic hypo vascularization of the pancreatic tissue is likely to be the cause of the presence of hypoxic areas within PDAC, which stimulate cancer cell to adapt their metabolism to the new microenvironment and have also been demonstrated to affect the efficacy of chemotherapy treatments [11,12,13].

## 3. Obesity and Pancreatic Cancer

The link between obesity (body mass index > 30.0 Kg/m^2^), type 2 diabetes, cardiovascular diseases and cancer has long been established, even though the mechanisms by which extra fat deposits increase cancer risk have yet to be fully elucidated. This is a dreary perspective considering that, according to the 2010 Health and Nutrition Examination Survey (NHANES), 35.5% of the USA adult population and 17% of children and teenagers are obese [14,15]. Pancreatic cancer is currently within the list of obesity-related cancers, together with colon, oesophageal, kidney, endometrial and postmenopausal breast cancer [15]. Obesity or a high-fat diet, is one of the factors that can increase the risk of developing acute pancreatitis [16,17], by changing the balance of digesting enzymes within acinar cells and lowering pancreatic enzyme secretion. Acute pancreatitis is characterized by an inflammatory state of the pancreas and by dysfunctional autophagy in pancreatic cells. In addition, by increasing the levels of the pro-inflammatory hormone leptin and decreasing the levels of the anti-inflammatory hormone adiponectin, obesity promotes inflammation. While normally inflammation is a natural response of the body, which activates immune cells using cytokines, chemokines and other mediators [18], persistent inflammation can lead to several cell damages caused by metabolic changes and oxidative stress. Similarly, obesity, promoting the activation of Akt and mTOR signalling pathways and down-regulating autophagy genes, such as Ulk1/Atg1 and Atg5, Atg6/Beclin1, inhibits autophagy, a cell defence mechanism which involves degradation and recycling of damaged cellular components and that controls inflammation [19]. Autophagy can also mediate mechanisms of chemoresitance of cancer cells to anticancer drugs. In response to metabolic and therapeutic stresses, autophagy induces cell death, increases inflammation and promotes tumorigenesis [20,21,22]. Moreover, the breakdown of excessive pancreatic fat caused by obesity produces a surplus of unsaturated fatty acids that can increase inflammation, parenchymal necrosis and lead to multi-organ damage and death [17]. Furthermore, unresolved or recurrent acute pancreatitis that shows a persistent low-grade inflammation can activate pancreatic stellate cells. These cells, which normally have the function of storing Vitamin-A lipid droplets in the cytoplasm, upon activation during pancreatic injuries, promote fibrogenesis, leading to chronic pancreatitis with an increased risk (around 5% of patients) of developing pancreatic cancer. In addition, the exceeding production of cytokines induced by the excessive number of immune cells stimulated by inflammation, can lead to the activation of oncogenic Kras, representing the initial switch for Kras activation followed by mutations to an oncogenic form, a typical feature of almost 90% of all pancreatic adenocarcinoma [19]. As well as many other cancer cells, one of the characteristic features of pancreatic cancer cells is the substantial alteration of cellular metabolism, which, together with genetic and epigenetic alteration, promotes tumour growth. In the early stages of cancer development, lipid biosynthesis, *de novo* lipogenesis, provides extra lipids necessary for the generation of biological membranes, energy store, and signalling functions [23]. It has been demonstrated that lipoprotein catabolism and cholesterol synthesis are very stimulated in PDAC, as tumour cells require high level of cholesterol [24]. Recent findings indicate that cancer cells can utilize diet-derived fats present in blood, together with *de novo* lipogenesis, to satisfy lipid necessities, strengthening the link between obesity, high fat diet, and cancer risk [25,26,27,28]. 

### 3.1. Epidemiological Studies

Recent epidemiological studies have also collected evidence supporting the connection between obesity and risk of pancreatic cancer. In the Metabolic Syndrome and Cancer Project, a study population of 577,315 individuals was observed for about 12 years follow-up and 315 women and 547 men were diagnosed with pancreatic cancer. As a result, a positive correlation between body mass index and risk of pancreatic cancer emerged, although only for women [29]. Results from a study conducted between 2008 and 2015 on 110 patients, where half of them were overweight or obese, showed a direct link between precancerous lesions of the pancreas, pancreatic fatty infiltration, intralobular fibrosis, subcutaneous and intravisceral fat and a high BMI. The authors also found the number of PanIN lesions to be correlated with the percentage of intravisceral fat, which was not found to be localized around the lesions. For this reason, they hypothesised fatty infiltrations to be the cause of PanIN lesions and not *vice versa* [30]. A large case-control study conducted in USA associated obesity to a 50%–60% elevated risk of developing pancreatic cancer, particularly amongst women and black subjects [31]. A positive association between pancreatic cancer risk and obesity and an increased risk, especially for women in the presence of waist localized fat, was also underlined by a pooled analysis from the National Cancer Institute Pancreatic Cancer Cohort Consortium (PanScan) [32]. A case-control study, conducted in the USA on 841 patients with pancreatic adenocarcinoma and 754 healthy individuals, found that obesity and overweight in early adulthood determined an increased risk of developing pancreatic cancer and led to precocity in the onset of this disease. Moreover, this study showed that survival rates of patients with pancreatic cancer were affected by the presence of obesity at an older age [33]. High BMI was also associated to poor survival in a retrospective study conducted on advanced or metastatic pancreatic cancer patients between 1994 and 2004 [34]. A pooled analysis of 14 cohort studies involving 846,340 persons found a 54% higher risk of pancreatic cancer for people obese at baseline and those who were overweight in their early adulthood. In addition, a 40% increased risk for individuals who had gained weight, compared to individuals who had a constant weight, and also an increase for persons with a high waist-to hip ratio was observed [35]. 

### 3.2. Experimental Studies

Experimental studies on animals also provided data to corroborate the connection between obesity and pancreatic cancer risk. Hamsters fed with a high fat diet and treated with *N*-nitrosobis(2-oxopropyl)amine (BOP) developed hyperlipidaemia and intrapancreatic fatty infiltration which led to pancreatic ductal adenocarcinoma in 67% of the animals, compared to 0% in the control group fed with a standard diet [36]. A study conducted on a genetically engineered mouse model revealed that mice fed with a HFD had an increased activation of oncogenic Kras via pro-inflammatory factor COX-2, compared to the control group. This resulted in an enhancement of the number of precancerous lesions of the pancreas and pancreatic ductal adenocarcinoma in the HFD group [37]. Taken together these data strongly support the theory that obesity and a high fat diet, by causing intrapancreatic fatty infiltration and promoting inflammation in the pancreas, trigger a chain reaction leading to the activation of oncogenic Kras signalling and to the developing of chronic pancreatitis and PanIn lesions, all well-known prerequisites of pancreatic cancer [38].

## 4. Diet and Pancreatic Cancer

Epidemiological and experimental studies have consistently shown the direct link between obesity, high BMI, weight gain and elevated risk of developing pancreatic cancer. As a consequence, it is logical to affirm that a high-calorie diet, and/or high consumptions of fats and sugars, predisposing over time to overweight or obesity, has a negative impact on pancreatic cancer risk. In addition, a high consumption of red meat has been found to elevate the risk of several types of cancer, including pancreatic cancer [39]. On the other hand, there is also evidence that a healthy diet can have a role in protecting against pancreatic cancer. In the 2010 Dietary guidelines for Americans (DGA), the recommendation focuses on maintaining a healthy weight through consuming the right amount of calories and nutrient-dense foods, together with the suggestion of eating a diet rich in fruit, vegetables and whole grains [5]. 

### 4.1. Phytochemicals and Dietary Fibre

It is well known that fruit, vegetables, whole grains and nuts contain elevated amounts of phytochemicals, bioactive compounds that can provide protection against several chronic diseases and cancer and that are classified as carotenoids, phenolics, alkaloids, nitrogen-containing compounds and organosulfur compounds [40,41,42,43]. Although their mechanisms of action are yet to be fully elucidated, phytochemicals have been discovered to possess an additive and synergistic action, which would account for their anticancer properties [44]. Some of the mechanisms proposed to explain phytochemicals anticancer properties include: antioxidant and anti-inflammatory action; inhibition of cell proliferation, differentiation, adhesion and invasion; anti-bacterial and anti-viral effects and stimulation of immune functions; DNA damage repair; regulation of steroid hormone and oestrogen metabolism; regulation of signal transduction pathways; enzyme regulation; inhibition of oncogene suppression and induction of tumour suppress gene expression; activation of cell cycle G arrest; induction of cell differentiation and apoptosis [44]. In addition to the role of phytochemicals, dietary fibre, one of the main components of fruit, vegetables, whole grains and nuts, has been found to have an inverse correlation with cancer risk. A case control study on 326 pancreatic cancer patients in Italy found soluble and insoluble fibre and fibre from fruit to be inversely associated to pancreatic cancer, even though no association between grain fibre and risk of pancreatic cancer was established [6]. On the contrary, whole grains were found to have a protective effect against several types of cancers, including pancreatic cancer, in a study on Mediterranean diet and cancer risk [39]. In the Nurses’ health study, a prospective study on 75,680 women, individuals who consumed a 28 g portion of nuts, two or more times per week were associated with a significant diminished risk of developing pancreatic cancer [45]. A case control study within EPIC cohort study (European Prospective Investigation into Cancer and Nutrition) recently reported an inverse correlation between plasma levels of beta-carotene (contained in orange fruit and vegetables and dark green leafy vegetables), zeaxantin (contained in paprika, corn and wolfberries) and alpha-tocopherol (contained in green and orange vegetables and tomatoes) and pancreatic cancer risk [46]. Moreover, some isothiocyanates, compounds contained in cruciferous vegetables (*i.e.*, broccoli, cauliflower, cabbage, and Brussels sprouts), such as sulforaphane, benzyl isothiocyanate and phenethyl isothiocyanate, have been shown to have an inhibitory effect on pancreatic cancer cells in *in vitro* and in animal studies [47,48,49,50,51]. 

### 4.2. Dietary Compounds and Autophagy

Autophagy can have different roles in cancer depending on tumour types and context [20]. Indeed, during the first stages of tumour progression autophagy prevents genomic instability and blocks tumour initiation, whereas in advanced states of the disease, autophagy, through the degradation and recycling of cellular components, contributes to the increased demand for rapid growth of cancer cells. PDAC are characterized by high levels of basal autophagy, and pharmacological or genetic suppression of autophagy have been shown to inhibit pancreatic cancer growth *in vivo* and *in vitro* [11]. Therefore, autophagy is required for tumour growth of PDAC, and drugs that inhibit this process have been proposed for clinical testing in PDAC patients, as well as in other tumour showing a similar dependence on autophagy. As a consequence of this increasing interest in targeting this process, there are currently several clinical trials involving autophagy inhibitors, such as chloroquine and its derivatives, worldwide. Among the different mechanisms through which dietary compounds can affect the risk of cancer, autophagy is emerging as an important process affected by diet [52]. This is not surprising considering that nutrient availability is the major regular of autophagy. Several dietary compounds have been shown to affect autophagy such as quercetin, genistein, curcumin, sulforaphane and resveratrol [53]. In addition, Vitamin D has been shown to influence autophagy and increasing evidence supports a potential role of vitamin D and its analogues in preventing or treating pancreatic cancer [54,55]. Nevertheless, several questions remain to be addressed, such as the dose and duration of exposures and tissue specificity in response to bioactive compounds as well as the implications of changes in autophagy during the early stages of tumour initiation.

### 4.3. Calorie Restriction

In addition to a diet rich in fruit, vegetables, nuts, and whole grains, calorie restriction is another promising strategy that seems to be effective in protecting against cancer risk [56]. In fact, by chronically controlling the amount of calories consumed (in animal model a 20% to 40% reduction is usually implemented), a reduction in the level of insulin, insulin-like growth factor, leptin, adiponectin, plasminogen activator inhibitor, cytokines and vascular endothelial growth factor is obtained. These changes contribute to lower inflammation and growth factor signalling, and to diminish vascular disorders, causing a reduction in cancer risk and cancer progression [56]. These findings clearly show a link between a high-calorie diet and the probability of developing pancreatic cancer and suggest a plan of action from a preventive perspective.

## 5. Conclusions

Given the extreme aggressiveness of pancreatic cancer and the difficulties in achieving an early diagnosis and an efficacious cure, it is mandatory to concentrate not only on finding new adequate treatments, but also on effective preventive strategies. As overweightness, high BMI and obesity are increasingly common in our society and can play a role in increasing the risk of pancreatic cancer, targeting these conditions and implementing a healthier lifestyle in the global population could be a method for having an impact in the prevention of pancreatic cancer, and also on other types of cancer and chronic diseases. A diet rich in fruit and vegetables, nuts and whole grain foods, together with a balanced calorie control, could be a valuable tool in future pancreatic cancer prevention strategies.
